# Mortality in older adults with severe mental illness: the role of metabolic syndrome and its components

**DOI:** 10.1192/j.eurpsy.2023.541

**Published:** 2023-07-19

**Authors:** S. Abou Kassm, M. Sánchez Rico, W. Naja, J. Alvarado, A. Halaby, F. Limosin, N. Hoertel

**Affiliations:** ^1^Centre Hospitalier Guillaume Régnier, Rennes; ^2^APHP, Issy-les-Moulineaux, France; ^3^Lebanese University, Beirut, Lebanon; ^4^Universidad Complutense de Madrid, Madrid, Spain

## Abstract

**Introduction:**

Studies in adult psychiatric patients consistently call attention to premature mortality and its association with metabolic syndrome. However, the utility of the metabolic syndrome construct is controversial in older adults in the general population, since literature shows that some components, such as obesity, can be protective against mortality. In older adults with mental illness, only one study explored the relation between metabolic syndrome and mortality and found no association.

**Objectives:**

To examine whether metabolic syndrome or any of its components predicted mortality in a cohort of older adults with psychiatric disorders, and to determine if this association differs across diagnostic groups.

**Methods:**

We used a multicentric prospective design to follow, over 5 years, a cohort that included 634 in– and outpatients with schizophrenia, bipolar or major depressive disorder (MDD). Metabolic syndrome was assessed at baseline following NCEP-ATPIII criteria. Cause of death was categorized as cardiovascular disorder (CVD) mortality, non-CVD disease-related mortality, suicide and accident.

**Results:**

We found no significant association between metabolic syndrome or any of its components with all-cause, CVD and non-CVD mortality. However, an association with increased all-cause and disease-related mortality was found in the subpopulation of older adults with MDD, even after adjustment for age, sex and smoking status (p=0.032 and p=0.036, respectively). A significant interaction was found between metabolic syndrome and psychiatric diagnoses indicating that in participants with MDD, metabolic syndrome had a significantly greater effect on all-cause mortality (p=0.025) and on disease-related mortality (p=0.008) than in participants with either bipolar disorder or schizophrenia.

**Conclusions:**

In older adults with psychiatric illness, our findings do not support an association between metabolic syndrome and increased mortality, in contrast with the literature findings on their younger counterparts. We discuss several possible explanations, including a survival bias, a lack of sensitivity of the used cut-offs and a ceiling effect of metabolic syndrome on mortality in this very high-risk population. The lack of a ceiling effect in the depressive subgroup, because of a less marked premature mortality, could explain the positive association, in contrast with bipolar disorder or schizophrenia subgroups.

**Disclosure of Interest:**

None Declared

